# Detection of slow port scans in flow-based network traffic

**DOI:** 10.1371/journal.pone.0204507

**Published:** 2018-09-25

**Authors:** Markus Ring, Dieter Landes, Andreas Hotho

**Affiliations:** 1 Faculty of Electrical Engineering and Informatics, Coburg University of Applied Sciences and Arts, 96450 Coburg, Germany; 2 Data Mining and Information Retrieval Group, University of Würzburg, 97074 Würzburg, Germany; Southwest University, CHINA

## Abstract

Frequently, port scans are early indicators of more serious attacks. Unfortunately, the detection of slow port scans in company networks is challenging due to the massive amount of network data. This paper proposes an innovative approach for preprocessing flow-based data which is specifically tailored to the detection of slow port scans. The preprocessing chain generates new objects based on flow-based data aggregated over time windows while taking domain knowledge as well as additional knowledge about the network structure into account. The computed objects are used as input for the further analysis. Based on these objects, we propose two different approaches for detection of slow port scans. One approach is unsupervised and uses sequential hypothesis testing whereas the other approach is supervised and uses classification algorithms. We compare both approaches with existing port scan detection algorithms on the flow-based CIDDS-001 data set. Experiments indicate that the proposed approaches achieve better detection rates and exhibit less false alarms than similar algorithms.

## Introduction

Company data are a valuable asset which must be protected against unauthorized access and manipulation [1]. Therefore, companies use various security mechanisms like firewalls or intrusion detection sytems (IDS) to protect their data [2]. Most of these operational security systems are, however, signature-based and cannot detect or prevent novel attack scenarios really well.

This work aims to support security experts and security systems in detecting novel and more serious attacks on the basis of data which are easy to obtain, while, at the same time, respecting the privacy of the user. To reach this goal, a flexible method is needed which is able to identify new attacks by utilizing and finally generalizing known behaviour. We take advantage of the fact that attack scenarios often follow a general sequence of phases. Skoudis and Liston [3] provide a widely known definition of five attack phases, namely *Reconnaissance*, *Scanning*, *Gaining Access*, *Maintaining Access* and *Covering Tracks*. In the *Scanning* phase, attackers often use port scans to identify hosts or networks which they want to infiltrate [4]. Normally, port scans trigger huge amounts of requests to different ports or IP Addresses within a short period of time. Such port scans can be easily detected by simple mechanisms like counting the number of requested ports for each *Source IP Address*. However, serious attackers scan their targets slowly in order to avoid suspicion. *Slow* means in this context that an attacker does not send probe packets permanently. Rather, attackers send probe packets to a host for example only every 15 seconds or every 5 minutes. Consequently, detection of slow port scans is more challenging. Due to the fact that scanning is an essential phase within a typical attack scenario, it is of upmost importance to detect slow port scans in order to identify new attacks. Detection of slow port scans must be taken into account for intrusion and insider threat detection.

This paper tackles the problem of detecting slow port scans in flow-based network data. Port scans as such do not cause any damage, but often constitute a forerunner of attacks that might cause serious harm. Thus, our work contributes to detecting attacks early, namely already in an initial stage during the *Scanning phase*.

Network flows provide meta information about network connections between endpoint devices. Information in flow-based data is significantly condensed in comparison to packet-based data. Hence, the detection of slow port scans is more complicated, but the amount of data to be analysed and, consequently, privacy concerns are reduced. Due to these advantages, we focus on flow-based data and propose two different approaches for the detection of (slow) port scans. The main idea of both approaches is to overcome the shortcomings of flow-based data by exploiting knowledge about the company network and characteristics of port scans in flow-based data. To that end, we propose an innovative preprocessing chain which is specifically tailored to detect slow port scans. We use knowledge about the company network to identify internal hosts. A flow describes only one specific connection whereas a port scan causes many connections. To overcome this issue, we collect flows over time windows. The collected flows are used to calculate new objects—which we call *network events*—with attributes like *number of flows directed to non-existing internal IP Addresses*. These *network events* are the basis of our two new port scan detection approaches. One approach is unsupervised and uses sequential hypothesis testing whereas the other approach is supervised and uses classification algorithms. Supervised approaches often achieve better results. Yet, in contrast to unsupervised approaches, they need labelled training data which are often hard to obtain. To generalize both approaches, we train and optimize them only on a subset of the flow-based data. Then, we apply these approaches to the remaining flow-based data to demonstrate how they perform on new data.

The paper’s main contribution is an innovative preprocessing chain which results in the generation of *network events* with attributes tailored for port scan detection. Further, we present two approaches for detecting (slow) port scans in flow-based network data.

The rest of the paper is organized as follows: the next section reviews related work on flow-based intrusion detection and on port scan detection algorithms. Then, the problem setting and the underlying flow-based data are discussed in more detail. Next, the preprocessing chain as well as approaches for detecting slow port scans are pesented. Furthermore, an experimental evaluation of both approaches is provided. The last section summarizes the paper and provides an outlook to future work.

## Related work

This work targets the detection of slow port scans in flow-based network traffic. Therefore, we provide an overview of flow-based intrusion detection before we review related work on port scan detection.

### Flow-based intrusion detection

Bhuyan et al. [5] provide an extensive review of packet- and flow-based anomaly detection methods. The authors categorize existing methods with respect to their technologies and categorize different attack types. In a more recent survey of data mining and machine learning for cyber security, Buczak and Guven [6] comprehensively review data mining methods for cyber security and provide an overview of well-known network-based data sets. Sommer and Paxson [7] identified various challenges (e.g. the lack of training data sets or the high cost of false alarms) for the use of anomaly-based network intrusion detection methods.

Packet- and flow-based network data consists of continuous and categorical attributes. Since categorical attributes have no natural order within their value ranges, standard similarity measures like the cosine similarity can not be used. The omnipresent IP Addresses in flow-based data are an example of categorical attributes. Many approaches deal with the similarity calculation of IP Addresses, e.g. Ring et al. [8] learned similarities between IP Addresses by extracting context information from flow-based network data. Weller-Fahy et al. [9] propose an overview of the used similarity measures of anomaly-based network intrusion detection systems. This work circumvents calculating similarities between categorical values by creating new objects (*network events*) with exclusively continuous attributes.

AlEroud and Karabatis [10] present a flow-based intrusion detection system that utilizes contextual information through semantic link networks (SLN). The authors extract time, location and other contextual information from flows to generate semantic links between alerts. Nychis et al. [11] propose an entropy-based anomaly detection approach which divides the flow-based data stream into five minute intervals and calculates various distributions for different attributes. Then, entropy values are calculated based on these distributions and used for anomaly detection. BClus [12] is an anomaly-based botnet detection method for flow-based data. It divides the flow-based data stream in time windows and aggregates the flows with respect to their *Source IP Addresses* in each time window. Then, new attributes are calculated for each aggregation and subjected to machine learning methods for botnet detection. While we aggreagte flow-based data streams like BClus [12], we compute new objects with adjusted attributes and focus on the detection of slow port scans rather than botnet detection as BClus does.

### Port scan detection

Over the years, considerable effort has been spent on network-based port scan detection algorithms. Network-based port scan detection algorithms may be divided into packet-based (*category I*) and flow-based approaches (*category II*).

Bhuyan et al. [13] provide an overview of port scanning and methodologies to the detection of port scans. The authors distinguish single source and distributed port scans and discuss a large number of port scan detection methods of both categories in detail. They summarize that most approaches are packet-based and that the lack of publicly available data sets complicates the evaluation and comparison of the different approaches [13]. Bou-Harb et al. [14] give a more recent review of port scanning. In particular, the authors categorize network scans according to their nature, strategy, and approach. Further, the authors explain different scanning methods and present existing methods for distributed port scan detection.

#### Packet-based port scan detection (category I)

Staniford et al. [15] present a packet-based detection algorithm for stealthy port scans from *category I*. The authors calculate an anomaly score for each packet which depends on the likelihood of occurrence for the *IP Addresses* and *Ports*. Anomalous packets are forwarded to *SPICE* (Stealthy Probing and Intrusion Correlation Engine). *SPICE* stores the packets as nodes in a correlation graph and uses thresholds to identify scanners. *Threshold Random Walk (TRW)* [16] is another packet-based port scan detection algorithm. *TRW* analyzes TCP packets only based on the assumption that port scanners have more failed connections than legitimate clients. TCP packets are distinguished as successful or failed based on TCP flags. *TRW* uses sequential hypothesis testing and defines two hypotheses *H*_0_ (the *Source IP Address* is normal) and *H*_1_ (the *Source IP Address* is a scanner). For each *Source IP Address* an accumulated ratio is updated after each arriving packet and checked against two thresholds. If the accumlated ratio exceeds any of the two thresholds, one hypotheses is accepted and the *Source IP Address* is marked as normal or scanner. In a nutshell, the approaches of Staniford et al. [15] and Jung et al. [16] rely on packet-based data whereas our approach focuses on flow-based network traffic.

#### Flow-based port scan detection (category II)

Sridharan et al. [17] transferred sequential hypothesis testing to flow-based data and present a representative of *category II*. The authors do not use a definition of successful or failed connections. Instead, their system *TAPS* collects the flow-based data over time windows and calculates for each *Source IP Address* the ratio of connected *Destination IP Addresses* and *Destination Ports*. The authors assume that port scanners have a very high or very low ratio in contrast to normal clients. If the ratio exceeds any of the two predefined thresholds, the *Source IP Address* is marked as *normal* or *scanner*. Further, Sridharan et al. [17] transferred the packet-based approach *TRW* to flow-based data and denominate it as *TRW-SYN*. *TRW-SYN* processes each flow separately and marks them as successful or failed based on the set TCP flags and number of packets. Zhang and Fang [18] propose *TFDS*, a port scan detection approach which is based on unidirectional flow-based data. *TFDS* is based on the observation that network scanners cause many small-sized flows and the flow size of normal traffic is larger and more variable in size. Therefore, *TFDS* collects the flow-based data over time windows and analyses the size and variation of the flows for each *Source IP Address*. For identifying port scanners, *TFDS* uses sequential hypothesis testing as *TAPS* and *TRW* do. The authors compared their method *TFDS* to *TAPS* and outperformed *TAPS*. Still, the high false positive rate is a weakness of both, *TAPS* and *TFDS*.

Gates et al. [19] use a Bayesian regression model to identify network scans at an Internet Service Provider (ISP) environment. Specifically, the authors collect flows for each *Source IP Address* over time periods and calculate objects with additional attributes as input for their model. Gates et al. [19] continue collecting flows until no further flow from the respective *Source IP Address* is received within a 5 minute time window. This may have been a valid assumption for ISP environments in the past. Nowadays, however, the permanent synchronisation of different services (e-mail clients, network drives, etc.) invalidates 5 minute time windows as appropriate stop criterion for company networks. Webster et al. [20] extended the list of calculated attributes of Gates et al. [19] and evaluated the performance of different classification algorithms for network scan detection in a university network.

Our approach does not process each flow separately like *TRW-SYN* [17], but resembles the approach of Gates et al. [19] in that it collects flows over times windows and calculates new objects with specific attributes. In contrast to the work of Gates et al. [19] and Webster et al. [20], our work utilizes knowledge about the company network. Thus, more specific attributes (e.g. *number of accesses to non-existing IP Addresses*) can be calculated which consider additional knowledge about hosts and network structures.

## Problem setting

Since this work is particularly directed towards identifying slow port scans in flow-based data, we first analyse port scans and flow-based data in more detail.

### Analysis of port scans

In active port scanning, an attacker identifies network hosts and services by transmitting probe packets. Many different port scan techniques exist and the behaviour of attackers and victims varies for these techniques. In the following, we focus on the main characteristics of port scan techniques. Port scanning can be broadly divided into horizontal and vertical scanning. Vertical scans are used to spot open ports of a single host while the more common horizontal scans are used to identify hosts for a specific open port [15].

#### TCP

The most common type of TCP port scanning is *SYN-Scan*. Here, the attacker sends the initial *SYN* request of the 3-Way-Handshake to the victim. Then, the victim sends a *SYN-ACK* response to the attacker if the port is open, or a *RST* response if the port is closed. In order to prevent log entries on the victim host, the attacker does normally not complete the 3-Way-Handshake for open ports. Therefore, this type of scan is often called *stealthy SYN-Scan* [15]. *FIN-Scans* are another TCP-Scan technique which sends *FIN* messages instead of *SYN* messages for bypassing firewall rules. If such a request is directed to a closed port, the victim sends a *RST* response. If an open port is addressed, the victim renders no response. Other TCP-Scan techniques are e.g. *ACK-Scans* or *XMAS-Scans* which send different combinations of TCP flags in the initial TCP request.

#### UDP

UDP scanning differs significantly from TCP scanning. If the attacker addresses an open UDP port, the victim does not necessarily render a response. However, similar behaviour can be observed when a firewall blocks the request. If the attacker sends the request to a closed UDP port, the victim normally responds with an *ICMP* unreachable message. Often, operating systems limit the number of *ICMP* unreachable messages to one per second [21].

In conclusion, observed flow-based network traffic varies significantly for different port scan techniques. The identification of victims seems to be easier than the identification of attackers since victims follow protocol rules and attackers vary their behaviour to trick security mechanisms. Further, port scan requests to closed ports seem to be easier to detect than those to open ports due to the abnormal behaviour of the victim.

### Flow-based data

Flow-based data provide meta information about network connections between endpoint devices and can be easily captured at network devices like switches. Claise [22] defines a flow as a sequence of packets with some common properties that pass through a network device. Traditionally, all packets which share the properties *Source IP Address*, *Source Port*, *Destination IP Address*, *Destination Port* and *Transport Protocol* are aggregated into one flow [23]. We use flows in NetFlow [22] format which follows this aggregation strategy. Port scans primarily aim at the identification of open ports on a specific host or at detecting various hosts with a specific open port. Since *Destination IP Address* and *Destination Port* are aggregation properties, a port scan causes a separate flow for each targeted host or targeted port.

Flows appear either in unidirectional or bidirectional format. Unidirectional flows represent a summary of all packets from host A to host B which share the same properties. The packets from host B to host A are aggregated in another unidirectional flow. In contrast, one bidirectional flow encompasses a summary of both, the transmitted packets from host A to host B as well as from host B to host A. Bidirectional flows contain more information but asymmetric routing in company backbones could distort the information in bidirectional flows [24]. Therefore, this work uses unidirectional flows in NetFlow [22] format.

*NetFlow* has an active and an inactive timeout for flows. The inactive timeout terminates a flow if no further packets are received within *α* seconds. The active timeout terminates a flow if it has been open for more than *β* seconds. By default, *NetFlow* uses the values *α* = 15 and *β* = 1800.

Table 1 shows typical attributes in unidirectional flow-based data.

**Table 1:** Typical attributes in flow-based data like *NetFlow* [22] or *IPFIX* [23].

#	Attribute	Description
1	Src IP	Source IP Address
2	Src Port	Source Port
3	Dest IP	Destination IP Address
4	Dest Port	Destination Port
5	Proto	Transport Protocol (e.g. ICMP, TCP, or UDP)
6	Date first seen	Start time flow first seen
7	Duration	Duration of the flow
8	Bytes	Number of transmitted bytes
9	Packets	Number of transmitted packets
10	Flags	*OR* concatenation of all TCP flags

## Detection approaches

In this section, we introduce our two approaches for detecting (slow) port scans. First, we propose the common idea of both approaches. Then, we present our novel preprocessing chain of flow-based data which results in the generation of *network events* tailored for port scan detection. Finally, we describe our two port scan detection methods *UPDS* (Unsupervised Port Scan Detection) and *SPDS* (Supervised Port Scan Detection).

### Outline of the proposed approaches

**Fig 1:**
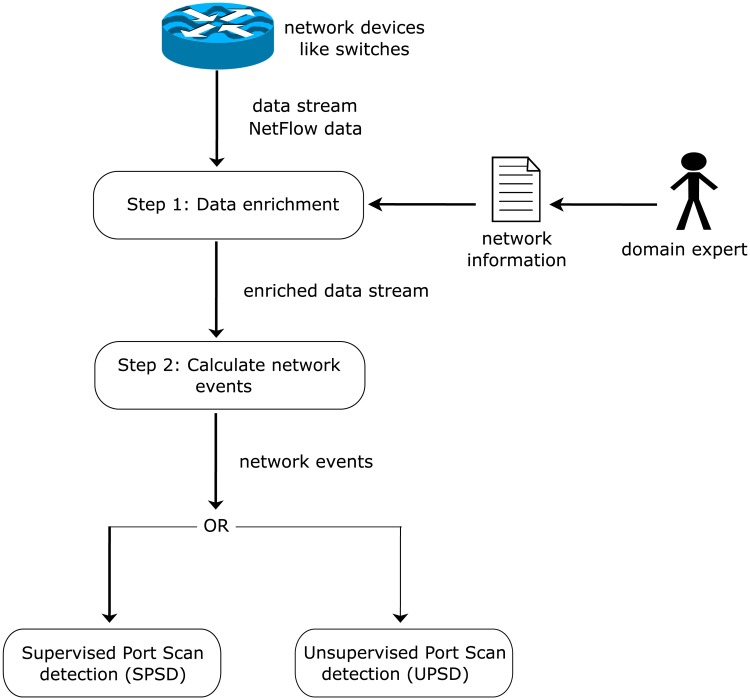
Proposed workflow for the detection of slow port scans.

Fig 1 provides an overview of the proposed approaches and illustrates the general workflow.

Starting point of both approaches is the unbounded flow-based data stream which is received from network devices like firewalls, routers or switches. In the first step, we use additional knowledge about the network structure to enrich the flow-based data stream with additional information. We tag the information if a *Source (Destination) IP Address* is internal or external to each flow. For this purpose, a so called *network information* file is required which needs to be set up by a domain expert and should contain all IP subnet addresses of the company network. After that, we collect the flows over time windows in the second preprocessing step. This helps us to confront the challenge that a port scan is characterized by sequences of flows and not by a single flow. The result of the second step is the generation of *network events*. These *network events* are used as input objects for our two port scan detection approaches. The first approach *UPSD* is unsupervised and uses sequential hypothesis testing. The second approach is supervised and uses classification algorithms. Both approaches process the incoming *network events* in real-time and depending on their results, port scan alerts are generated or not.

### Preprocessing of flow-based data

Our preprocessing chain (Step 1 and Step 2 in Fig 1) results in the generation of *network events*. Therefore, we collect all incoming flows within a time window of *δ* seconds. Then, we calculate for each *Source IP Address* in each time window one *network event* based on these collections. These *network events* are used as input values for our detection algorithms *UPSD* and *SPSD*.

#### Generation of network events

Attackers use port scans to collect information about a network. Their goal is to learn about open ports which they can exploit in succeeding steps. Consequently, it is more likely for attackers to address closed ports or non-existing *IP Addresses* than for legitimate clients when scanning the whole network for open ports. We use this heuristic to build *network events* with appropriate attributes for port scan detection. Further, we exploit additional knowledge about the network structure. More specifically, we store all internal *IP Addresses* and any known open TCP port for internal *IP Addresses*. The latter is achieved by recording any combination of *Source IP Address* and *Source Port* (between 0 and 1024) for which a valid TCP connection could be observed. Table 2 provides a summary of the calculated attributes for *network events*.

**Table 2:** Attributes within a *network event*.

#	Name	Description
1	IP	IP Address
2	ICMP-Error count	The number of received ICMP Errors
3	RST count	The number of received RST flags from different *targets*
4	RwA count	The number of addressed *targets* without response
5	NeIP count	The number of flows to non-existing internal IP Addresses
6	NeTCP count	The number of flows to non-existing internal TCP-Services
7	Succession count	Counts how often this *IP Address* had a value greater than 0 in one the other attributes in succession

#### Example

In the following, we want to explain the attributes of *network events* through an example (see Tables 3 and 4). Table 3 shows the known internal *IP Addresses* and known open TCP ports. Table 4 provides an overview of the captured flows within an exemplary time window. The first attribute of the *network event* is the *IP Address* of the host. The *IP Address* is only used to identify the source of *port scans* and is not considered by further analysis methods. In our example, we want to create a *network event* for the host with the IP Address *192.168.220.16*. A unique combination of *Destination IP Address* and *Destination Port* is called a *target*.

**Table 3:** The table delineates known internal IP Addresses and known open TCP ports for the exemplary network.

Known internal IP Addresses	Known open TCP ports
192.168.100.5	192.168.100.5 Port 80
192.168.100.6	192.168.100.6 Port 22
192.168.100.15	192.168.100.5 Port 443
192.168.220.16	

**Table 4:** The table contains the collected flows for a specific time window for the exemplary network.

#	Proto	SrcIP	SrcPt	DstIP	DstPt	TCP flags
1	TCP	192.168.100.5	80	192.168.220.16	53321	.A….
2	TCP	192.168.220.16	53333	192.168.100.5	80	.A..S.
3	TCP	192.168.100.5	80	192.168.220.16	53333	.A..S.
4	TCP	8.8.8.8	80	192.168.220.16	47898	…R..
5	TCP	192.168.100.5	80	192.168.220.16	53333	…R..
6	TCP	192.168.100.5	80	192.168.220.16	53321	…R..
7	TCP	192.168.220.16	53337	192.168.100.5	22	….S.
8	TCP	192.168.220.16	53338	192.168.100.5	22	….S.
9	TCP	192.168.220.16	53339	192.168.100.5	23	….S.
10	TCP	192.168.100.5	22	192.168.220.16	53337	…R..
11	TCP	192.168.100.5	22	192.168.220.16	53338	…R..
12	TCP	192.168.100.5	23	192.168.220.16	53339	…R..
13	TCP	192.168.220.16	53340	192.168.100.15	443	….S.
14	TCP	192.168.220.16	53341	192.168.100.15	443	….S.
15	TCP	192.168.220.16	53342	192.168.100.15	443	….S.
16	TCP	192.168.220.16	53343	192.168.100.15	22	….S.
17	ICMP	192.168.220.16	0	192.168.220.44	8.0	……
18	ICMP	192.168.220.1	0	192.168.220.16	3.1	……
19	UDP	192.168.220.16	34345	192.168.100.5	53	……
20	ICMP	192.168.100.5	0	192.168.220.16	3.3	……

The attributes 2 and 3 for *network events* (see Table 2) consider the identified behaviour of victims (see Section *Analysis of Port Scans*). The attribute *ICMP-Error count* contains the number of received ICMP unreachable flows and is an indicator for UDP scans. For our example, the value of *ICMP-Error count* is 2, since the *IP Address 192.168.220.16* receives two ICMP unreachable messages (see flow number #18 and #20) in Table 4.

Similarly, the attribute *RST count* is an indicator for TCP scans. If an attacker addresses a closed TCP Port, the victim normally sends a RST flag as response. The attribute *RST count* tries to measure this behaviour. However, RST flags are also used to cancel existing connections. Therefore, we only consider flows with received RST flags from different *targets* for which a previous request and no established connection could be observed. The value of *RST count* in our example is 2 (flows #10, #11 and #12). Flows #10 and #11 only count as one, since they are received from the same *target*. There is no request from *192.168.220.16* to *8.8.8.8*. Therefore, the RST flag of flow #4 is not considered. The RST flags of flows #5 and #6 are also not counted since an open connection for these flows can be observed (see flows #1 and #3).

Often firewalls and other security mechanisms block probe packets of attackers. In these cases, we can observe a unidirectional flow from the attacker to the victim even if the probe packets never reach their targets. In consequence, the victim does not return a response and, hence, no corresponding unidirectional flow from the victim to the attacker can be observed, which is reflected in the fourth attribute, *RwA count*. This attribute contains the number of flows without corresponding flows in the opposite direction. Flows with multicast or broadcast addresses in the attribute *Destination IP Address* are excluded by this counter. Multiple unidirectional flows to the same *target* without corresponding counterpart in the opposite direction are only counted once. The value of *RwA count* is 4 in our example. Here, we count the flows #13, #14, #15, #16, #17 and #19. However, the flows #13, #14, #15 have the same *target* and are only counted once.

As already mentioned above, it is more likely for attackers to address closed ports or non-existing *IP Addresses* than for legitimate clients. The attributes 5 and 6 try to consider these facts. The fifth attribute *NeIP count* contains the number of requested non-existing internal *Destination IP Addresses*. In our example, all *IP Addresses* with *192.168.X.X* are internal. Consequently, the value of *NeIP count* is 1 (flow #17).

Further, we store all known open TCP ports for internal hosts. The attribute *NeTCP count* contains the number of flows to different *targets* which address non open known TCP ports on internal hosts. In our example, the attribute *NeTCP count* has the value 3 (flows #7, #8, #9 and #16). Flows #7 and #8 together only count once.

For detecting slow port scans, we use the following approach. The attributes *ICMP-Error count*, *RST count*, *RwA count*, *NeIP count* and *NeTCP count* are indicators for port scans. If all indicator attributes are zero, we set the value of attribute *Succession count* to 0. Otherwise, we distinguish the following two cases: (1) The value of the attribute *Succession count* for this *IP Address* was zero in the previous time window. In this case, we set the value of the attribute *Succession count* to 1. (2) The value of the attribute *Succession count* for this *IP Address* had value *v* in the previous time window. In this case, *Succession count* will be set to *v* + 1. Consequently, the attribute *Succession count* contains the information how often the *IP Address* has a value greater than 0 in any indicator attribute in succession. This heuristic is based on the assumption that badly configured clients may exceed the threshold for a short period, but only port scanners do so over longer periods.

### UPSD—Unsupervised port scan detection

In the unsupervised *UPSD* approach, we transfer Jung’s idea of sequential hypothesis testing [16] for port scan detection to our *network events* outlined in the previous section.

Sequential hypothesis testing updates a likelihood variable with a given stream of events in order to assign this stream to one of two possible sets *H*_0_ or *H*_1_. In our scenario, the stream of events consists of *network events* from the same *Source IP Address*. Further, the set *H*_0_ contains the normal hosts and the set of port scanners is described by *H*_1_. Each *network event* is represented as an indicator variable *Y*_*i*_:

$$\begin{array}{c} Y_{i}=\{\begin{array}{cc} 0 & \text{if}\;\text{network}\;\text{event}\;\text{i}\;\text{is}\;\text{sucessful}\\ 1 & \text{if}\;\text{network}\;\text{event}\;\text{i}\;\text{is}\;\text{not}\;\text{sucessful} \end{array}, \end{array}$$ (1)

Therefore, we have to define if a *network event* is successful or not successful. Since our primary goal is a low number of false alarms when detecting slow port scans, we choose only strong indicators for port scans from Table 2. We experimentally observed that the attributes *RST count* and *RwA count* have often values greater than 0 for normal network traffic. One reason for this observation is the processing of the data stream which divides the data stream into fixed time windows. Let us assume, the unidirectional flow from host A to host B appears in time window *t* and unidirectional flow from host B to host A (response) appears in time window *t* + 1. Consequently, the attribute *RwA count* would be 1 for host A in time window *t* and 1 for host B in time window *t* + 1. For this reason, we only consider the attributes *ICMP-Error count*, *NeIP count* and *NeTCP count*. We compute the sum *α* of these three attributes. If *α* is 0, the *network event* is successful, otherwise it is not successful. Since sequential hypothesis testing considers times series from scratch, it makes no sense to consider the attribute *Succession count*.

Further, there are four probabilities attached to the indicator variable *Y*_*i*_:

$$\begin{array}{c} Pr\left[Y_{i}=0|H_{0}\right]=\theta_{0} \end{array}$$ (2)

$$\begin{array}{c} Pr\left[Y_{i}=1|H_{0}\right]=1-\theta_{0} \end{array}$$ (3)

$$\begin{array}{c} Pr\left[Y_{i}=0|H_{1}\right]=\theta_{1} \end{array}$$ (4)

$$\begin{array}{c} Pr\left[Y_{i}=1|H_{1}\right]=1-\theta_{1} \end{array}$$ (5)

Eqs 2, 3, 4 and 5 describe the probabilities that an event is successful (or not successful) on condition that it belongs to the set *H*_0_ (or *H*_1_). It is important to note that there must be a significant difference between the statistical behaviour of *H*_0_ and *H*_1_. For example, the set of normal hosts *H*_0_ should have more successful *network events* than the set of port scanners *H*_1_. The probabilities *θ*_0_ and *θ*_1_ are user-defined input parameters. For each incoming *network event*, the sequential hypothesis testing updates the likelihood ratio of the corresponding *Source IP Address* as follows:

$$\begin{array}{c} ratio_{i}^{srcIP}=ratio_{i-1}^{srcIP}*\alpha_{i}*\frac{Pr[Y_{i}=\gamma |H_{1}]}{Pr[Y_{i}=\gamma |H_{0}]} \end{array}$$ (6)

where *γ* is 0 for successful *network events* and 1 for not successful *network events*. The variable *α*_*i*_ is the sum of the attributes *ICMP-Error count*, *NeIP count* and *NeTCP count* from the recent *network event* *i*. If *α*_*i*_ is 0 (successful *network event*), we set the value of *α*_*i*_ to 1 in order to prevent a multiplication by 0.

After updating the ratio, we check it against two thresholds *η*_0_ and *η*_1_. If $ratio_{i}^{srcIP}$ exceeds any of the two thresholds, the *Source IP Address* will be labelled with the corresponding class and the ratio of this *Source IP Address* is reset to 1. If $ratio_{i}^{srcIP}<\eta_{0}$, then the *Source IP Address* could be assigned to *H*_0_ (normal host). If $ratio_{i}^{srcIP}>\eta_{1}$, then the *Source IP Address* could be assigned to *H*_1_ (scanner). In this case, *UPSD* raises an alert that the *Source IP Address* is a port scanner.

Algorithm 1 shows the pseudo code of *UPSD*.

**Algorithm 1**: Unsupervised algorithm *UPSD* for detecting slow port scans.

*srcIPRatios* // HashMap for storing the ratios

**while** end of network event stream is not reached **do**

 *event* = read next *network event*

 *ip* = *event*.*srcIP*

 **If** ip not in srcIPRatios **then**

  *srcIPRatios*.*add*(*ip*, 1.0)

 **end**

 *ratio* = *srcIPRatios*.*get*(*ip*)

 *α*_*i*_ = *event*.*ICMP*-*Error*_*count*+*event*.*NeIP*_*count*+*event*.*NeTCP*_*count*

 **if** *α*_*i*_ > 0 **then**

$ratio=ratio*\alpha_{i}*\frac{1-\theta_{1}}{1-\theta_{0}}$

 **else**

$ratio=ratio*1*\frac{\theta_{1}}{\theta_{0}}$

 **end**

 *srcIPRatios*.*put*(*ip*, *ratio*)

 **If** *ratio* > *η*_1_ **then**

  mark *ip* as scanner

  *srcIPRatios*.*put*(*ip*, 1.0)

 **end**

 **If** *ratio* < *η*_0_ **then**

  mark *ip* as normal

  *srcIPRatios*.*put*(*ip*, 1.0)

 **end**

**end**

### SPSD—Supervised port scan detection

Our supervised *SPSD* approach is based on classification algorithms. To be precise, we use *Support Vector Machines (SVM)* and *Decision Trees*. Generally, classification algorithms are used to assign objects to different predefined groups (classes). The process of classification consists of two steps. In the first step, the classification algorithm is trained with a labelled set of training objects. Each training object contains a label indicating the class the object belongs to. The classification algorithm learns the characteristics of the different classes and builds a model or function for the assignment (classification) of new or unlabelled objects. In the second step, the classification algorithm uses this model or function to classify unseen objects [25]. The *network events* are the input objects in our port scan scenario and we have two classes: *normal* and *attack*. We use the attributes #2, #3, #4, #5, #6 and #7 (see Table 2) of *network events* for training the classification algorithms. After training the classification algorithm, we use it to classify unseen *network events*. If a *network events* is assigned to the class *attack*, *SPSD* raises an alert that the corresponding *Source IP Address* is a port scanner.

#### Decision tree

A decision tree builds a tree to classify input objects and has three basic elements. Every inner node checks the value of an attribute. The edges of the inner nodes represent the possible results of the attribute check. Depending on the attribute values, a certain path is followed until a leaf node is reached. The leaf nodes assign class labels to objects and do not perform further comparisons.

In the training phase, the decision tree is learnt from the data. Starting at the root node, an attribute is assigned to the node. The algorithm selects the attribute which optimizes an evaluation function. For every possible attribute value, a child node is created and the training objects are separated into sets according to their value of this particular attribute. This process is repeated for each child node, until all training objects belong to the same class or until all attributes have already been assigned to inner nodes on this path. Normally, a pruning step is performed after the decision tree has been created. Pruning reduces the complexity of the tree by removing unnecessary branches and improves classification accuracy [26].

#### Support vector machines

SVMs are binary classifiers for linear and non-linear data which classify input objects into two different classes. Often, SVMs use a kernel function which transforms the input data to a higher-dimensional space, the so-called *feature space*. In this *features space*, SVMs search for the optimal hyperplane which separates the input objects with respect to their classes. The optimal hyperplane maximizes the distance to the nearest objects on both sides.

In the classification phase, a SVM maps the new input objects to the *feature space* and uses the identified hyperplane to classify them [26].

## Experiments and discussion

This section presents an experimental evaluation of our proposed port scan detection approaches (*UPSD* and *SPSD*) on the flow-based CIDDS-001 data set [27]. We compare our approaches with the widespread flow-based port scan detection algorithms *TRW-SYN* [17] and *TFDS* [18]. Further, we use an adjusted version of Webster et al. [20] as third baseline since it seems to be the most similar approach to our work.

### Evaluation data set

We use the CIDDS-001 (https://www.hs-coburg.de/cidds) [27] data set for evaluation. In the CIDDS-001 data set, a small business environment was rebuilt using the software platform *OpenStack* and the generated network traffic was captured in unidirectional *NetFlow* format. The small business environment contains about 20 clients and typical servers like *File-Server* and *Email-Server*. The user behaviour of the clients is simulated by scripts which execute typical user activities on the clients. The scripts take restrictions such as working hours and different working styles into account in order to generate network traffic that is as realistic as possible. Further, the authors executed several attacks within the network and labelled all flows.

The CIDDS-001 [27] data set consists of two parts. One part contains the flow-based network traffic within the *OpenStack* environment. The other part contains flow-based network traffic observed at an external server in the internet. In this work, we use only the *OpenStack* part of the data set. This part contains 4 weeks of network traffic where the first two weeks contain several attacks and the last two weeks are free of attacks. For our experiments, we use the first two weeks of this data set and refer to them as *week1* and *week2*.

We edited the data set as follows. At first, we filtered out all attacks except scanning attacks. The remaining data set contains only normal traffic and scanning attacks. The flows can be distinguished in three classes: *attacker*, *victim* and *normal*. Since we are only interested in identifying attackers, we changed the label *victim* to *normal* for all flows. The resulting data set contains more than 15 millions flows which can be distinguished in *normal* and *attacker*. Table 5 provides an overview of the executed port scans within this data set.

**Table 5:** Port scans within the CIDDS-001 data set.

Parameter	week1	week2
T = 1	7	6
T = 2	10	7
T = 3	9	1
Sum	26	14

The authors of [27] used the tool *nmap* for the execution of port scans within the CIDDS-001 data set. Table 5 shows that there are 26 port scans in *week1* and 14 port scans in *week2*. The parameter T controls the timing behaviour of the port scans: the higher the value of *T*, the faster the port scan. A port scan with parameter *T* = 1 sends a probe packet every 15 seconds whereas a port scan with parameter *T* = 2 sends a probe packet every 0.4 seconds [21].

Other existing labelled data sets like *ISCX* [28] or MAWI repository [29] are not suitable for our evaluation setting. The MAWI repository [29] publishes up-to-date packet-based data sets which are captured at an internet backbone. Using these data sets would destroy the fundamental idea of the proposed approaches since it would be impossible to integrate additional knowledge about the network structure. In contrast to that, the flow-based data set ISCX [28] does not focus on port scans, but rather on various other types of attacks such as *(Distributed) Denial of Service*. Thus, it cannot serve as a reasonable baseline for our purpose.

### Evaluation methodology

We use the number of recognized scanning attacks and the number of false alarms as evalulation measures.

*TRW-SYN* processes each flow separately and assigns each flow to the class *normal* or *attacker*. *TFDS*, *Webster*, *UPSD* and *SPSD* collect flows over time windows and assign each collection to the class *normal* or *attacker*. Due to this collection procedure, some collections can contain *normal* and *attacker* flows (we refer to them as *mixed collections* in the following). *Mixed collections* can not be uniquley assigned to the class *normal* or *attacker*. Consequently, we have to use additional rules for labelling *mixed collections*. In this work, we assign each *mixed collection* to the class *attacker*. Therefore, the classification accuarcy would not be a fair evaluation measure, since not all approaches (*TRW-SYN*) use this additional labelling rule. For this reason, we do not use the classification accuracy as additional evaluation measure.

### Parameter setting

This section provides information about the parameters settings of the different approaches. Further, it should be considered that our approach *SPSD* and the work of *Webster* are supervised methods which need labelled data in the training phase. In contrast to that, *TRW-SYN*, *TFDS* and *UPSD* are unsupervised methods which do not need labelled data in the training phase.

#### TRW-SYN

TRW-SYN is controlled by four user-defined input parameters. We evaluate *TRW-SYN* with two different parameter settings. First, we use the parameter from [17] and refer to them as *TRW-SYN (default)*: *η*_1_ = 99, *η*_0_ = 0.01, *θ*_1_ = 0.2, and *θ*_0_ = 0.8. Then, we empirically optimized the parameters for *week1* of the CIDDS-001 data set. We refer to this parameter setting as *TRW-SYN (optimized)*: *η*_1_ = 99999, *η*_0_ = 0.0001, *θ*_1_ = 0.3, and *θ*_0_ = 0.6.

#### TFDS

TFDS needs the input of seven user-defined parameters. Here, we also evaluate two different parameter settings. First, we use the parameter from [18] as default parameter *TFDS (default)*: *η*_1_ = 256, $\eta_{0}=\frac{1}{256}$, *θ*_1_ = 0.2, *θ*_0_ = 0.8, *c* = 10, *α* = 0.1 and *t* = 20. Second, we empirically optimized the parameters for *week1* of the CIDDS-001 data set. We refer to this parameter setting as *TFDS (optimized)*: *η*_1_ = 10000, $\eta_{0}=\frac{1}{10000}$, *θ*_1_ = 0.25, *θ*_0_ = 0.75, *c* = 10, *α* = 0.15 and *t* = 40.

#### UPSD

*UPSD* collects all incoming flows within a *δ* second time window and calculates for each *Source IP Address* within this time window one *network event*. We set the parameter *δ* = 60 for the calculation of time windows.

Further, this approach needs like *TRW-SYN* four user-defined input parameters. We configured the parameter such that we detect the maximum number of port scans in *week1* of the CIDDS-001 data set. We refer this setting as *UPSD (maxDect)*: *η*_1_ = 99, *η*_0_ = 0.01, *θ*_1_ = 0.2, and *θ*_0_ = 0.8. Then, we tried to reduce the number of false alarms for *week1*. We refer this setting as *UPSD (minFP)* and used the following configuration: *η*_1_ = 999, *η*_0_ = 0.001, *θ*_1_ = 0.2, and *θ*_0_ = 0.8.

#### SPSD

Similar to *UPSD*, we set the window parameter *δ* = 60 for the calculation of time windows. Then, we use *Decision Tree* and *SVM* classifiers for the identification of port scans. These classifiers have to be trained with labelled training data before they can be used to classify new *network events*. Therefore, we trained the classifiers on *week1* to classify *week2* and vice versa. All continuous attributes of *network events* are normalized through scaling to the interval [0, 1]. Both classification algorithms have some configurable parameters. For *SVM*, we used a *RBF* kernel with parameters *C* = 10000 and *γ* = 1.0. For *Decision Tree*, we used the *GINI* index as split criterion.

#### Webster

Webster et al. [20] collect the flows for each *Source IP Address* and calculate aggregations with additional attributes. The authors apply an attribute selection algorithm and discretize all attributes. Based on these preprocessing steps, the authors evaluate the performance of different classification algorithms. For defining the third baseline—which we call *Webster* -, we calculate the same attributes as [20], use the Random Forest classifier which obtained the best results in [20] and set the parameter *lower bound* to 1. However, we make an adjustment for our evaluation. Webster et al. [20] collected the flows for each *Source IP Address* over the complete data set. As we try to detect port scans in (nearly) real time, it would not be appropriate to collect the flows of a *Source IP Address* over the complete data set. Hence we split the data stream in time windows of 60 seconds (like for *USPSD* and *SPSD*) and calculate one aggregation for each *Source IP Address* within each time window. Similar to *SPSD*, we train the Random Forest classifier on *week1* to classify *week2* and vice versa.

### Results

Table 6 shows the detection rate and false alarms of *UPSD*, *SPSD*, *TRW-SYN*, *TFDS* and *Webster* on the CIDDS-001 data set.

**Table 6:** Results of the experiment. The table shows the number of detected port scans as well as the number of false alarms for the different algorithms in *week1* and *week2*. *Week1* contains 26 port scans and *week2* contains 14 port scans.

Approach	week1		week2	
	Detected attacks	False alarms	Detected attacks	False alarms
TFDS (default)	6	35	2	16
TFDS (optimized)	9	29	4	8
TRW-SYN (default)	15	1685	8	7495
TRW-SYN (optimized)	14	78	7	118
UPSD maxDect	**26**	1	**14**	2
UPSD minFP	25	**0**	**14**	**0**
SPSD with DecisionTree	**26**	**0**	**14**	8
SPSD with SVM	**26**	**0**	**14**	**0**
Webster	**26**	22	**14**	6

*TFDS* has the lowest detection rate of port scans. We are able to increase the detection rate and simultaneously decrease the false alarm rate through parameter optimization for *TFDS*. However, it only detects 9 out of 26 attacks in *week1* and 4 out of 14 in *week2*. In contrast to that, *TRW-SYN* is able to detect more port scans than *TFDS*. We are able to reduce the number of false alarms significantly by customizing the user input parameters. However, this approach still generates the most false alarms. Depending on the parameter configuration, *UPSD* is able to recognize all port scans (*UPSD (maxDect)*) or to generate no false alarms (*UPSD minFP*). The proposed supervised approach *SPSD* is able to detect all 26 port scans in *week1* and all 14 port scans in *week2*. Further, *SPSD with SVM* has no false alarms for both weeks. While the other supervised algorithm *Webster* is able to detect all port scans in *week1* and *week2*, it generates in contrast to *SPSD* a larger number of false alarms than *SPSD*.

### Discussion

Table 6 shows that *SPSD* reaches the best results. It has the highest detection rate (all port scans were detected) of port scans combined with the lowest rate of false alarms. We used *Decision Tree* and *SVM* classifiers as detection algorithms. Both classification algorithms have their advantages. *Decision Trees* build classification models which are human readable. Each path from the root node to a leaf node defines a classification rule. In comparison to that, the *SVM* classifier achieves the lowest rate of false alarms.

The supervised approach *Webster* is also able to detect all port scans for *week1* and *week2*. However, it generates a higher number of false alarms than *SPSD* and *UPSD*.

Our unsupervised approach *UPSD* reaches the second best result. For the first parameter setting (see *UPSD (maxDect)* in Table 6) it is able to recognize all attacks along with a low false alarm rate (1 false alarm in *week1* and 2 false alarms in *week2*). For the second parameter setting (see *UPSD (minFP)* in Table 6) it is able to generate no false alarms and simultaneously detect 25 of 26 port scans in *week1* and all port scans in *week2*.

*TRW-SYN* is limited to the detection of TCP SYN scans only. Since the CIDDS-001 data set also contains other types of port scans, the detection rate of this approach is lower than the detection rate of the proposed approaches. Further, the flows in the CIDDS-001 data set are captured with *OpenVSwitch*. *OpenVSwitch* has an inactive timeout of 1 or 2 seconds for flow generation (depending on the version of *OpenVSwitch*). In comparison to that, the default inactive timeout of *NetFlow* in real hardware devices is 15 seconds. The shorter inactive timeout of the flows within CIDDS-001 data set may lead to more *failed* connections for *TRW-SYN*. Therefore, we tried to customize the parameters of this approach to reduce the effect of *failed* connections (see *TRW-SYN (optimized)* in Table 6). Through this parameter tuning, we were able to reduce the number of false alarms enourmouly.

*TFDS* has the lowest detection rate of port scans, see Table 6. A more detailed analysis showed that *TFDS* was not able to detect slow port scans. Further, it has also problems to detect port scans from attackers which try to cover their traces. For example, *TFDS* is not able to detect the port scans when the attacker simultaneously performs legal activities like surfing the web.

Overall, *SPSD* has the highest detection rate combined with the lowest rate of false alarms. Further, *SPSD* is not limited to some types of TCP Scans like *TRW-SYN*, instead it is able to detect various types of slow port scans. However, *SPSD* needs like *Webster* correctly labelled training data to learn a classification model. Such data sets are rare, but can be generated by manual labelling or simulation (see e.g. [27, 28], or [30]). In contrast to that, *UPSD* reaches nearly the same results and does not need any training data. Instead, this approach has some user-defined parameters which must be tuned manually. The same holds for *TRW-SYN* and *TFDS*. However, these approaches have lower detection rates and more false alarms compared to the proposed *UPSD* approach.

## Conclusion

Attacks against company networks often pass the typical attack phases: *Reconnaissance*, *Scanning*, *Gaining Access*, *Maintaining Access* and *Covering Tracks*. Normally, attackers use port scans in the preliminary *Scanning* phase to gather information about their targets. For those reasons, recognition of port scans can be an early indicator for future attacks. However, the detection of slow port scans is challenging due to the large amount of network traffic in company networks.

In this paper, we propose two approaches for detecting slow port scans within flow-based network data. Both approaches use the same innovative preprocessing chain which is also the main contribution of this work. This preprocessing chain collects for each host flow-based network traffic over time windows and enriches the flows with additional information about the network structure (e.g. is the *Source IP Address* internal or external). Then, the enriched and collected flows are used to calculate new objects—which we called *network events*—with appropriate attributes for port scan detection. These *network events* are used as input for our detection approaches: *UPSD* (Unsupervised Port Scan Detection) and *SPSD* (Supervised Port Scan Detection). *UPSD* uses sequential hypotheses testing whereas *SPSD* is based on classification algorithms. Both approaches reduce the amount of data due to the transformation of flows to *network events* which simultaneously minimizes the analysis effort for security experts.

We evaluated our approaches experimentally on the CIDDS-001 data set. The results indicate that both approaches are able to detect slow port scans with a very low false alarm rate. In the future, we intend to expand the evaluation of the proposed approaches with real world network data and upcoming evaluation data sets. Further, we want to investigate the usage of additional domain knowledge in other attack phases.
